# The radioenhancer NBTXR3: bridging physics and biology to improve radiotherapy outcomes and prime anti-tumor immunity

**DOI:** 10.1186/s13046-025-03579-1

**Published:** 2025-12-17

**Authors:** Célia Bienassis, Omar I. Vivar, Yun Hu, Jordan Da silva, Audrey Darmon, Julie Marill, Naeemunnisa Mohamed Anesary, Peter Schmitt, Laurent Levy, James Welsh, Yolanda Prezado, Frédérique Megnin-Chanet, Sébastien Paris

**Affiliations:** 1 Nanobiotix, Paris, France; 2 Department of Radiation Oncology, The University of Texas MD Anderson Cancer Center, Houston, USA; 3 Institut Curie, Université PSL, CNRS UMR3347, Inserm U1021, Signalisation Radiobiologie et Cancer, Orsay, France; 4 Université Paris-Saclay, CNRS UMR3347, Inserm U1021, Signalisation Radiobiologie et Cancer, Orsay, France; 5 INSERM U 1196/CNRS UMR 9187, Paris-Saclay Research University, Orsay, France; 6 Institut Curie, Bat. 112, Rue H. Becquerel, Orsay, France

**Keywords:** Antitumor immunity, Cancer, Nanoparticle, NBTXR3, Proton therapy, Radioenhancer, Radiotherapy

## Abstract

Radiotherapy remains a cornerstone in cancer treatment, used in over 50% of cases. It employs ionizing radiation, primarily X-rays, to target and destroy tumors through direct DNA damage and indirect effects via reactive oxygen species. Despite technological advancements improving precision of the delivered dose to the tumor, radiotherapy faces critical challenges, particularly damage to healthy tissues, which limits the maximum safe dose. Recent years have seen significant improvements in radiation delivery, including advanced imaging for real-time tumor tracking and combinations with immunotherapy. However, the need for innovative strategies to enhance radiotherapy’s therapeutic index remains essential. The radioenhancer NBTXR3 could represent a solution in addressing these limitations. This nanotechnology has been designed to amplify radiotherapy’s effects within tumors without increasing toxicity in non-injected adjacent healthy tissues. Beyond better cancer cell destruction and tumor control, radiotherapy-activated NBTXR3 nanoparticles can also stimulate systemic antitumor immune responses in preclinical models. This review aims to provide a comprehensive analysis of preclinical research on NBTXR3, focusing on its mechanism of action and role in initiating and enhancing antitumor immune responses.

## Background

### Current challenges in radiotherapy

Radiotherapy (RT) has been a cornerstone in the treatment of solid tumors for decades. Today, at least 50% of cancer patients undergo radiotherapy as part of their treatment regimen [1]. RT primarily employs ionizing radiation (IR), most commonly X-rays, to selectively target and destroy cancerous lesions. The therapeutic efficacy of RT hinges on its ability to induce physical interactions between IR and cellular components, which result in molecular alterations to DNA, lipids, proteins, and other critical structures within the cell. The antitumor effects of RT arise from both direct and indirect mechanisms of cellular damage. Direct interactions between ionizing radiation and DNA can generate double-strand breaks (DSBs), which represent the most lethal form of DNA injury and can trigger cell cycle arrest, senescence, or diverse cell death pathways, including apoptosis, necrosis, mitotic catastrophe, autophagy, and ferroptosis [2]. However, under normoxic conditions, the majority of radiation-induced damage—approximately 70%—results from indirect effects [3]. These are mediated by the radiolysis of intracellular water, leading to the production of reactive oxygen species (ROS) such as hydroxyl radicals and hydrogen peroxide. ROS further amplify DNA and cellular damage, ultimately enhancing tumor cell killing. The interplay between direct ionizing radiation damage and ROS-driven oxidative stress forms the basis of RT’s therapeutic efficacy.

Despite significant technological advances in recent years, RT continues to face critical challenges that limit its full therapeutic potential. The introduction of more sophisticated linear accelerators and imaging systems has undoubtedly improved the precision and efficacy of RT, allowing for better tumor targeting and reduced side effects [4]. However, a key limitation remains: the damage to healthy neighboring tissues that are exposed to radiation while targeting the tumor. This collateral damage restricts the maximum dose of radiation that can be safely administered to patients, thus constraining the overall effectiveness of the treatment. To address these limitations, there is a growing need for innovative strategies that can enhance the therapeutic index of RT. To this end, radioenhancers such as gold nanoparticles [5] and other high-Z element-based platforms have been explored [6, 7]. Radioenhancer nanoparticles share the common goal of amplifying the effects of ionizing radiation by increasing local energy deposition and the generation of reactive oxygen species (ROS). Despite differences in composition, size, and surface functionalization, all radioenhancer nanoparticles require efficient tumor accumulation and cellular internalization to achieve therapeutic efficacy.

NBTXR3 is a novel nanoparticle that enhances the effects of RT without impacting the surrounding non-injected health tissue, thereby widening the therapeutic ratio [8, 9]. The purpose of this review is to provide a comprehensive analysis of the preclinical research on NBTXR3 nanoparticles.

### Main text

#### NBTXR3

**Fig. 1:**
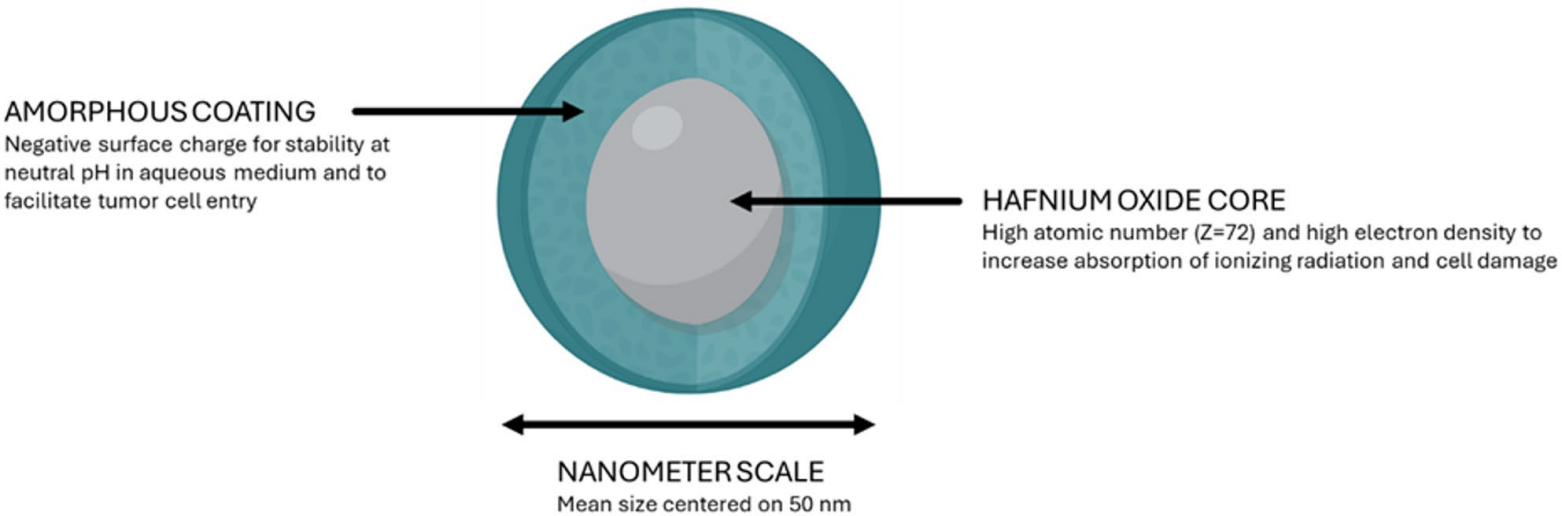
Schematic representation of the composition of a nanoparticle of NBTXR3

NBTXR3 is a novel therapeutic for solid tumors delivered through intratumoral injection (IT) and used in combination with RT [10]. NBTXR3 is radioenhancer, composed of a core of functionalized hafnium oxide (HfO_2_) which has a high atomic number of (Z = 72), and functionalized at an average size of 50 nm while being covered with a negative phosphate surface charge (Fig. 1).

**Fig. 2:**
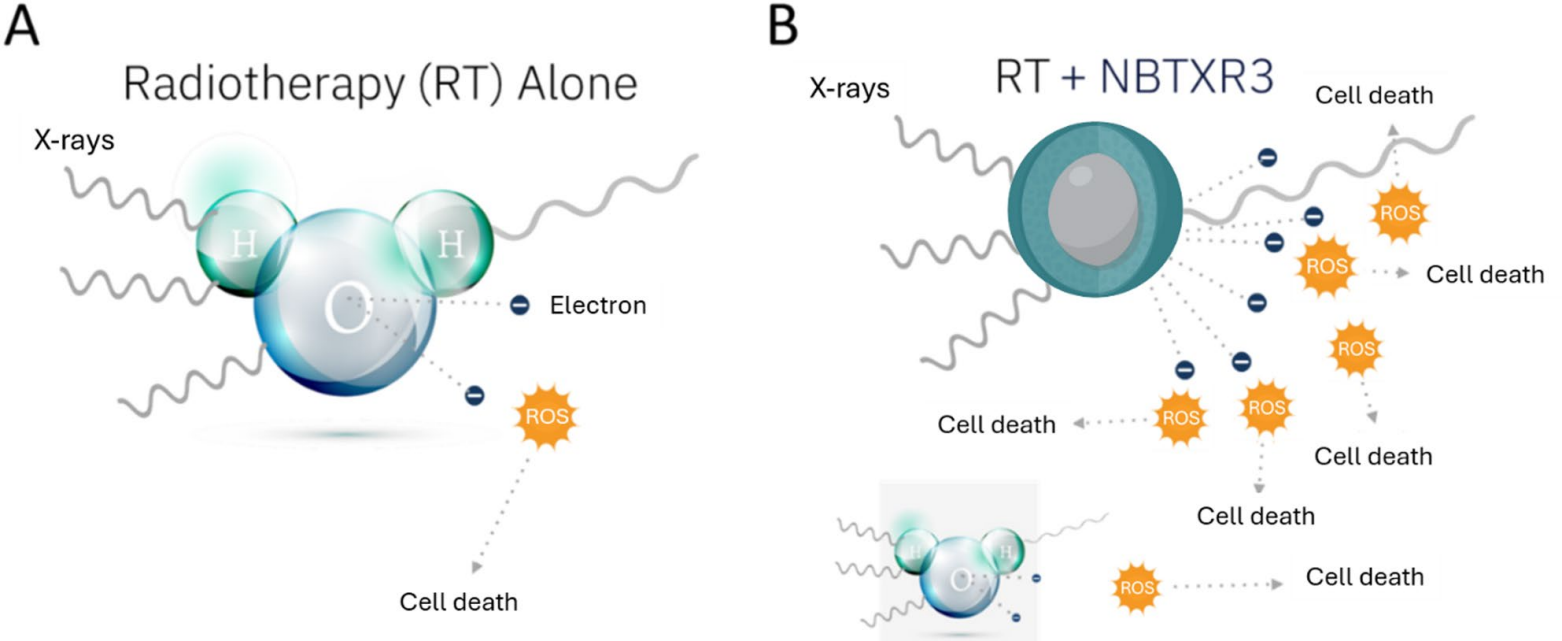
Principle of the physical mode of action of NBTXR3. Schematic representation of ROS generation between **A** a water molecule and **B** a NBTXR3 nanoparticle

The therapeutic effect of NBTXR3 is based on the interaction of ionizing radiation with the nanoparticles (Fig. 2). The high electron density of hafnium increases the likelihood of interaction with IR, thereby enhancing the energy dose deposited within cells. When exposed to RT, NBTXR3 boosts the production of ROS, intensifying the damage to cancer cells without increasing the radiation dose. This approach allows for enhanced radiation effects within the tumor without impacting the surrounding non-injected healthy tissues [8, 9, 11, 12].

#### Entry into cells and intracellular fate

**Fig. 3:**
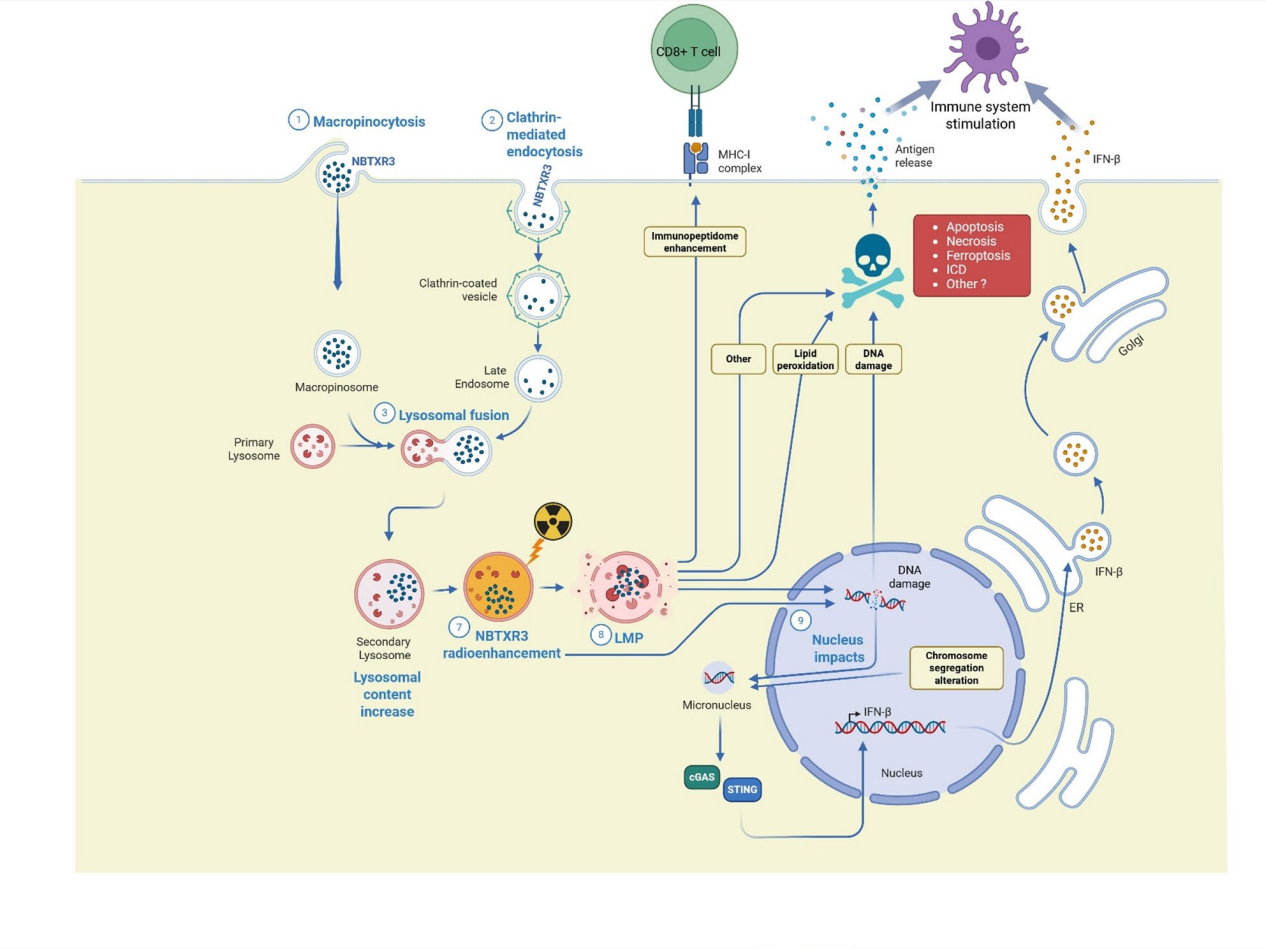
Model presenting the biological responses induced by NBTXR3 alone or activated by radiotherapy

Transmission electron microscopy (TEM) is the only technique that allows direct visualization of NBTXR3 nanoparticles within cells. NBTXR3 is radiopaque and can therefore be visualized on CT scan to control its distribution within the tumor. Maggiorella et al. [13] utilized TEM both in vitro and in vivo to demonstrate that NBTXR3 nanoparticles are internalized by tumor cells and accumulate in the cytoplasm, forming clusters of nanoparticles. Marill et al. [14] confirmed uptake in multiple human cell lines, including Hs913T (fibrosarcoma), HT-29 (colorectal adenocarcinoma), 42-MG-BA (glioblastoma), and PANC-1 (pancreatic carcinoma). Additional studies have documented NBTXR3 internalization in 16 different human and mouse cell lines [13–17] (Table 1). No nanoparticles were detected in the nucleus in any cell line at any concentration. Da Silva et al. [18] conducted in vitro experiments that analyzed the kinetics of endocytosis. The authors reported that the amount of NBTXR3 inside the cells was directly related to the concentration used. Endocytosis mechanisms were also analyzed, using TEM on HT1080 (human fibrosarcoma), 42-MG-BA, and CT26.WT (mouse colon carcinoma) cell lines at intervals of 1, 3, and 24 h after NBTXR3 addition. Results indicate that the predominant entry route was macropinocytosis nanoparticles appeared in vesicles in the cytoplasm within an hour, clustering into larger groups over time (Fig. 3).

**Table 1:** Compilation of preclinical studies conducted with NBTXR3. The numbers indicated in the in vitro and in vivo columns correspond to the numbers of the associated bibliographic references

Tissue origin	Cancer type	Cell line name	Species	In vitro		In vivo	
				TEM	Efficacy	µCT	Efficacy
Brain	Glioblastoma	42-MG-BA	Human	[13, 14, 18]	[14]		
		T98G	Human		[17]		
Breast	Adenocarcinoma	MDA-MB-231-luc-D3-H2LN	Human	[17]			
		TSA	Mouse				[23]
Colon	Carcinoma	CT26	Mouse	[18]	[20]	[20]	[20]
		HCT116	Human	[13, 14]	[14, 24]	[13, 14]	[13, 14]
	Adenocarcinoma	HT29	Human	[14]	[14]		
Head and Neck	Tongue squamous cell carcinoma	CAL-33	Human	[17]	[14]	[17]	[17]
	Pharyngeal squamous cell carcinoma	Detroit 562	Human	[17]			
	Hypopharyngeal squamous cell carcinoma	FaDu	Human	[17]	[14, 17]	[17]	[17]
Liver	Hepatocellular carcinoma	HEP-3B	Human		[17]		
Lung	Large cell lung carcinoma	NCI-H460-Luc2	Human	[17]	[14, 17]	[17]	[17]
	Adenocarcinoma	344SQR	Mouse	[16]		[16]	[16]
Lung metastasis	Fibrosarcoma	Hs913T	Human	[14]	[14]		
Pancreas	Carcinoma	MIA PaCa-2	Human		[17]		
	Ductal adenocarcinoma	PANC-1	Human	[14]	[14]		
Prostate	Carcinoma	DU-145	Human	[17]	[17]	[17]	[17]
	Adenocarcinoma	LNCaP	Human	[17]			
		PAC-120	Human			[17]	
		PC-3	Human	[17]	[17]	[17]	[17]
Soft tissue	Ewings Sarcoma	A673	Human				[13, 14]
	Fibrosarcoma	HT1080	Human	[13, 14, 18]	[13, 14]		[17]
	Liposarcoma	LPS80T3	Human			[17]	[17]

Endosomes formed through macropinocytosis usually fuse with lysosomes [19]. Immuno-TEM analysis confirmed that NBTXR3 nanoparticles are indeed present in these organelles. By using NBTXR3^RED^ (NBTXR3 labeled with dextran tetramethylrhodamine), researchers observed that most clusters were co-localized with lysosomes 16–24 h after being added to 42-MG-BA, HCT116 (human colon carcinoma), HT1080, and CT26.WT cells [18]. Interestingly, starting 16 h after NBTXR3 addition, there was a marked increase in the mean fluorescence intensity (MFI) of the LysoTracker signal in CT26.WT, 42-MG-BA, HT1080, and HCT116 cell lines, suggesting an increase in lysosome numbers. This possibility is consistent with the transcriptome analysis of CT26.WT cells, showing a global increase in the expression of lysosome-related genes, including those for lysosomal membrane proteins, acidification enzymes, hydrolases, lysosome formation, and transport, 24 h after the addition of NBTXR3. Similar results were measured in vivo in the CT26.WT model 24 h after intratumoral injection. Overall, these results indicate a shared mechanism for nanoparticle entry, and intracellular trafficking, across all tumor cells tested.

#### Distribution and retention in tumors

To assess distribution, several in vivo micro-computed tomography (µCT) scan studies evaluated the dispersion and retention of NBTXR3 in various human tumors engrafted in nude mice. Maggiorella et al. [13] first demonstrated that NBTXR3 was detectable in the HCT116 cell line for at least 14 days after IT. Zhang et al. [17, 20] and Hu et al. [16] studied NBTXR3 localization the day after IT and at various time points across different mouse models. Their findings showed that the nanoparticles were well distributed within the tumor mass the day after injection. µCT scans performed one to two weeks later confirmed that the nanoparticles remained localized within the tumor. Notably, in the patient derived xenograft (PDX) PAC-120 model, known for its very slow tumor growth, nanoparticles were still present 50 days after the initial µCT scan [20]. Endobronchial ultrasound-guided injection of NBTXR3 into hilar and mediastinal lymph nodes, as performed by Casal et al. in swine, demonstrated that the procedure can be performed safely, achieving a high rate of nanoparticle retention, minimal extravasation, and no visible nanoparticle embolization [21]. Overall, these results suggest long term retention of NBTXR3 within tumors after injection. Similar tumor retention has been seen clinically across a range of tumor types (sarcoma [8, 11, 12], rectal cancer [22], and head and neck cancer [9]).

#### Evaluation of efficacy

##### In vitro efficacy

The initial demonstration of the benefits of NBTXR3 + RT to enhance cancer cell death, compared to RT alone, was achieved through clonogenicity tests conducted on HT1080 cells [13]. These first results were confirmed and expanded upon in other cell lines by Marill et al. [14] and Zhang et al. [17], using the same approach. Currently, the benefits of NBTXR3 + RT, compared to RT alone, have been demonstrated in 16 different cell lines, reflecting a diversity of cancer types (such as glioblastoma, prostate, liver, lung, sarcoma, pancreatic, and breast cancers) [13, 14, 17, 20, 23] (Table 1). In each case, NBTXR3 + RT improved efficacy compared to RT alone, while NBTXR3 alone does not impact cell survival. However, some cell lines appear to be more sensitive to the treatment than others. This variability could be due to their intrinsic radioresistance, lower internalization of NBTXR3, or other factors not yet identified. However, in all studied cell lines, combining NBTXR3 with increasing concentrations or doses of RT led to a measurable, dose-dependent increase in cell death and dose enhancement factor (DEF) compared to RT alone [13, 14, 17, 24].

##### In vivo efficacy

Several studies using cancer cell lines subcutaneously injected into mice were performed to confirm that NBTXR3 + RT can more effectively destroy tumor cells than RT alone [13, 17, 23] (Table 1). When combined with RT, NBTXR3 significantly controlled tumor growth and improved survival across all models, compared to RT alone. This combination notably increased tumor doubling time and median survival, while NBTXR3 alone does not impact tumor growth. In the radioresistant DU-145 prostate tumor model, RT alone had no significant effect on tumor growth. In contrast, treatment with NBTXR3 + RT resulted in a substantial delay in tumor growth, increasing the tumor doubling time from 9 days with RT alone to 20 days. These findings are consistent with data published for the A673 model (human Ewings Sarcoma) [13]. Overall, the advantages of RT-activated NBTXR3 for local tumor growth delay were demonstrated in 11 different models.

### Fate of NBTXR3 nanoparticles released after cell death

It was hypothesized that after tumor cell destruction, released NBTXR3 nanoparticles could be taken up by surviving tumor cells. In vitro microscopy and flow cytometry analyses using 42-MG-BA-GFP human glioblastoma cells and NBTXR3^RED^ nanoparticles confirmed the re-endocytosis of nanoparticles from dead cells. In the experiments conducted by Da silva et al. [18], NBTXR3^RED^ nanoparticles were first added to wild-type 42-MG-BA cells. After 16 h of incubation, 42-MG-BA cells containing NBTXR3^RED^ were sorted by FACS to eliminate non-endocytosed NBTXR3^RED^ nanoparticles and then returned to culture. These cells were either irradiated to induce cell death (or left untreated), and subsequently co-cultured with 42-MG-BA-GFP cells. NBTXR3^RED^ nanoparticles were not detected in 42-MG-BA-GFP cells when co-cultured with unirradiated 42-MG-BA cells, but were observed when co-cultured with irradiated cells, indicating that nanoparticles released from dead cells can be recaptured by surviving cancer cells. TEM analyses show that the recaptured nanoparticles formed clusters similar to previously described studies, indicating the same biological processes may occur in the same way, even in these challenging conditions.

These results indicate that NBTXR3 released by dead cells can be taken up by surviving tumor cells, increasing their intracellular concentration and making them more sensitive to destruction during subsequent RT sessions. This creates a cycle of enhanced tumor cell destruction and nanoparticle recapture. This may explain, at least in part, the notable persistence of these nanoparticles within tumor tissues.

### Early mechanisms of cell death induction

Cell death is the final outcome of a series of preceding biological processes. Some early biological events that can trigger cell death and may be amplified by NBTXR3 + RT have been studied.

#### DNA damage

An essential feature of effective RT is its ability to induce DNA damage, particularly DSBs. RT disrupts DNA integrity, impairing cancer cell replication and survival. If unrepaired, DSBs lead to cell death and are thus a key target in cancer treatment. Marill et al. [24] investigated whether the enhanced cell death observed with NBTXR3 + RT could be associated with increased DSB formation. To test this, the authors analyzed γ-H2AX staining by flow cytometry, 30 min post-RT in HCT116-DUAL cells. The percentage of γ-H2AX positive cells was significantly higher in NBTXR3 + RT treated cells compared to RT alone (11.2% ± 0.38 vs. 7% ± 0.6, *p* < 0.001 at 2 Gy; 24.4% ± 1.07 vs. 16.9% ± 0.29, *p* < 0.0001 at 4 Gy). This study reveals that NBTXR3 + RT can increase DSB formation, but the underlying mechanisms responsible for the enhanced DSB generation induced by this treatment remain to be fully established.

A corollary to the formation of DSBs is the generation of micronuclei (MN). These small, extranuclear bodies detected in the cytoplasm form when chromosome fragments or whole chromosomes fail to be properly incorporated into the daughter nuclei during cell division [25]. This can occur because of errors in DNA repair following DSBs, leading to chromosomal instability. Both RT and NBTXR3 + RT significantly increased MN formation in a dose-dependent manner compared to untreated cells [24]. However, NBTXR3 + RT induced significantly more MN than RT alone (7.1% ± 0. 3 vs. 4.2% ± 0.29, *p* < 0.001 at 2 Gy; 16.1% ± 0.67 vs. 12.8% ± 0.51, *p* < 0.0001 at 4 Gy), indicating that NBTXR3 + RT is more effective than RT alone at promoting MN formation in HCT116-DUAL cells [24].

#### Induction of lysosomal membrane permeabilization

Lysosomes are critical organelles which play a role in degrading macromolecules, calcium regulation, and other fundamental cellular functions [26]. Maintaining their integrity is crucial for cell health. Under stress, lysosomal membrane permeabilization (LMP) can release enzymes like cathepsins into the cytosol, triggering cell death [27]. Given NBTXR3’s accumulation in lysosomes, the potential impact of these radiotherapy-activated nanoparticles on these organelles have been investigated in CT26.WT, HT1080, 42-MG-BA, and HCT116 cell lines [18]. LysoTracker analyses showed that RT alone did not reduce the signal, indicating no LMP. In contrast, NBTXR3 + RT led to significant signal loss in all cell lines tested indicating LMP. Immunofluorescence microscopy analyses confirmed cathepsin D release in all cells treated with NBTXR3 + RT, which was not observed in cells treated with RT alone.

Lipid peroxidation plays a central role in ferroptosis by inducing oxidative damage to cell membranes, ultimately leading to membrane destabilization and cell death [28]. BODIPY (a sensitive fluorescent probe used to measure lipid peroxidation in live cells and membrane systems) revealed a significant increase in lipid peroxidation in NBTXR3 + RT treated cells compared to RT alone. This strongly suggests enhanced cell death by ferroptosis. In CT26.WT, HT1080, 42-MG-BA, and HCT116 cells, lipid peroxidation rose by approximately 39%, 33%, 36%, and 39%, respectively. These studies demonstrate that NBTXR3 + RT uniquely induces LMP, leading to increased lipid peroxidation and enhancing cell death pathways by ferroptosis beyond what RT alone achieves. This highlights a possible distinction between the biological responses triggered by RT alone and those achieved with NBTXR3 + RT.

### Cell death analysis

The early biological events induced by NBTXR3 + RT can differ from RT alone in both their nature (e.g., LMP) and intensity (e.g., DSBs and MN). This suggests these differences could potentially influence the types of cell death that occur. To determine whether NBTXR3 + RT leads to an increase in the same types of cell death as RT, or if these nanoparticles can alter and/or modulate the nature of the cell death types, different cell states (viability, early apoptosis, early necrosis, and late apoptosis/necrosis) were measured 48 h after treatment in CT26.WT cells [20]. For cells treated with NBTXR3 alone, the viability was the same as for untreated control cells. Compared to RT alone, treatment with NBTXR3 + RT significantly decreased cell viability by increasing early apoptosis, early necrosis, and late apoptosis/necrosis. The effect of NBTXR3 + RT was particularly notable for early apoptosis at 2 Gy. Interestingly, the reduction in cell viability with NBTXR3 + 4 Gy was greater than with 6 Gy RT alone, suggesting the radioenhancement capability of NBTXR3. In the CT26.WT cell line, RT alone appears to primarily induce early apoptosis in a dose-dependent manner. In contrast, necrosis does not seem to be a main cell death pathway, as the percentage of cells in early necrosis remained modest and constant across the tested radiation doses. However, for NBTXR3 + RT there was a dose-dependent increase in the percentage of cells undergoing early necrosis. Even if the percentage remains relatively modest compared to apoptosis, this suggests that the addition of NBTXR3 to RT influences the induction of this specific cell death phenotype, leading to a greater proportion of cells dying via early necrosis compared to RT alone. The dose-dependent increase in early apoptosis with RT alone, and the shift towards more early necrosis with NBTXR3 + RT, suggest that the combination treatment can modulate the predominant cell death pathways compared to RT monotherapy in this CT26.WT cell line model. Further investigation would be needed to fully elucidate the mechanisms behind these differential effects on cell death types.

In an independent study using the HCT116-DUAL cell line, it has also been reported that NBTXR3 + RT provided benefits over RT alone in terms of reducing cell viability [24]. However, the impact on the type of cell death differed from the previous study using CT26.WT cells. In this study, HCT116-DUAL cells treated with NBTXR3 + RT showed a significant increase in early necrosis markers compared to RT alone, but there were no significant changes in markers of early apoptosis across all conditions tested.

### Beyond cancer cell destruction: modulation of adjuvanticity and immunogenicity of cancer cells

The release of antigens by dead tumor cells is crucial for the activation of the antitumor immune response [29]. When tumor cells die, they release various tumor-associated antigens into the surrounding environment. These antigens are then taken up by dendritic cells and other antigen-presenting cells, which process and present them to T cells [30, 31]. This process stimulates the immune system to recognize and attack remaining tumor cells, enhancing the effectiveness of cancer treatments and contributing to long-term immune surveillance against cancer recurrence. NBTXR3 + RT, by inducing more cell death than RT alone, has the potential to enhance this antitumor immune response. Indeed, the enhancement of cell death triggered by NBTXR3 + RT leads to the release of more tumor-associated antigens, which can better stimulate the antitumor immune system to target and eliminate cancer cells more effectively. Other factors preceding cell death could also play a crucial role in activating the antitumor immune response. Multiple preclinical studies have shown that the combination of NBTXR3 + RT can impact various pathways associated with antitumor immune activation, boosting the immune system’s capacity to identify and destroy tumor cells [15, 16, 20, 32].

#### cGAS-STING activation

NBTXR3 + RT significantly increases MN formation. Free DNA found in the cytoplasm is a powerful trigger for the cGAS-STING pathway [33, 34]. This cascade ignites a robust immune response by stimulating type I interferon production, particularly interferon-beta. The resulting immune activation is multifaceted: it enhances antigen presentation, mobilizes natural killer cells, and promotes cytotoxic T lymphocyte activation and differentiation [35]. Essentially, this pathway acts as a crucial bridge between radiation-induced cellular damage and a potent anti-tumor immune response. Marill et al. [24] demonstrated that NBTXR3 + RT significantly amplifies cGAS-STING pathway activation in HCT116-DUAL cells. These engineered cells, containing a luciferase reporter gene responsive to interferon signaling, provide a sensitive readout of pathway activation. At radiation doses ranging from 1 to 4 Gy, NBTXR3 combined with radiotherapy (RT) consistently outperformed RT alone, increasing luciferase activity by 30–60%. This enhanced pathway activation was further corroborated in an independent study using TSA cells (mouse breast adenocarcinoma), where NBTXR3 + RT triggered markedly increased interferon secretion [23].

#### Immunogenic cell death

Immunogenic cell death (ICD) is a form of cell death that triggers an immune response by releasing damage-associated molecular patterns (DAMPs) and tumor antigens, which can activate dendritic cells and other immune cells [36–38]. The ICD involves the translocation of calreticulin to the cell surface (ecto-CALR), the secretion of ATP and the release of high-mobility group box 1 (HMGB1), which collectively act as “eat me”, “find me” and “activate me” signals for immune cells, respectively [38]. RT has been shown to induce ICD by causing cellular stress and damage that lead to the exposure of these immunogenic signals [39–42]. This mechanism enhances the immunogenicity of tumors and stimulates the antitumor immune response. NBTXR3 + RT impact on ICD biomarkers was assessed across multiple cell lines (HCT116, 42-MG-BA, CT26.WT, and PANC-1) [32]. For ecto-CALR analysis, cells were treated with NBTXR3 overnight then irradiated. Twenty four hours after RT, cells were processed using specific antibodies and analyzed by cytofluorometry. As expected, RT alone significantly increased ecto-CALR in all cell lines, with fold increases ranging from 1.7 to 2.7-fold. NBTXR3 + RT showed even higher increases: 2.4 to 7.9-fold. Other ICD markers analyses (extracellular ATP and HMGB1 release) also showed increased levels with NBTXR3 + RT treatment.

#### Modulation of immunopeptidome

The immunopeptidome consists of small protein fragments (peptides) displayed on cell surfaces by MHC class I molecules [43]. These peptides, derived from intracellular proteins, act as cellular “fingerprints” that can be recognized by CD8 + T cells through their T-cell receptors (TCR). In cancer, tumor cells often display altered peptides due to mutations or abnormal protein expression. When a CD8 + T cell’s TCR specifically recognizes a tumor-derived peptide presented by MHC-I, it triggers T cell activation and a targeted immune response against the cancer cells [32]. This TCR-peptide-MHC interaction is fundamental for anti-tumor immunity.

Darmon et al. [32] showed the impact of NBTXR3 + RT on tumor immunogenicity. Using CT26.WT cells, they analyzed MHC class I-associated peptides under various treatment conditions. While NBTXR3 alone induced minimal changes and RT alone elevated 33 peptide levels, the NBTXR3 + RT combination dramatically amplified peptide abundance. Compared to untreated cells, 155 peptides showed a two-fold increase in abundance, representing 46.8% of all peptides analyzed—approximately 4.7 times more than with RT alone. The origin of these peptides revealed that NBTXR3 + RT generated a more diverse peptide pool compared to RT alone. While nuclear proteins dominated in both cases, NBTXR3 + RT triggered peptides from the cytoskeletal, plasma membrane, and lysosomal proteins, broadening the antigenic landscape. Most importantly, NBTXR3 + RT significantly enhanced the immunogenic potential of the peptides, increasing those with positive immunogenicity scores. This suggests a potential more robust activation of CD8 cytotoxic T-cell-mediated antitumor responses. Although the precise mechanisms await further investigation, this study suggests the capacity of NBTXR3 + RT to reshape the cancer cell immunopeptidome.

### Modulation of the antitumor immune response

The combination of biological responses involved in the antitumor immune reaction—including DNA damage, cGAS-STING pathway activation, ICD, and modulation of the immunopeptidome—is notably enhanced with NBTXR3 + RT. This enhanced immune response has the potential to trigger an abscopal effect, a phenomenon where localized RT treatment leads to the reduction or elimination of tumors at distant, untreated sites, likely due to systemic immune activation [44]. Zhang et al. [20] tested this hypothesis in vivo using a dual-flank tumor mouse model with CT26.WT cell tumors. The growth curves showed that RT alone slowed treated tumor growth, but no abscopal effect was seen as untreated tumor growth matched the control. In the group of mice treated with NBTXR3 + RT, the growth of the treated tumor was also slowed, similar to the RT-only group. However, this group exhibited a significant abscopal effect, with untreated tumors also responding and median survival extended. To better understand how this phenomenon takes place, the authors analyzed the densities of CD4 + T, CD8 + T, and CD68 + macrophage cells in both tumors. For CD4 + cells, only the irradiated tumors showed a noticeable change compared to control groups, with no significant difference between RT and NBTXR3 + RT, although NBTXR3 + RT showed a slight trend of increase. In a separate study using a single-tumor model, Darmon et al. [32] reported a significant rise in CD4 + cell infiltrates observed in mice treated with NBTXR3 + RT, compared to the RT group. Significant increases in CD8 + and CD68 + cells were found in both treated and distant untreated tumors with NBTXR3 + RT, compared to RT. CD8 + cells were notably more abundant in both tumor types with NBTXR3 + RT. This finding was confirmed in a second study [32]. To assess the role of CD8 + cells in the abscopal effect induced by NBTXR3 + RT, a two-tumor experiment was repeated with mice depleted of CD8 + cells before treatment. Results in non-depleted mice matched previous findings, showing a strong abscopal effect with NBTXR3 + RT. But when CD8 + cells were depleted, the abscopal effect disappeared, underscoring their essential role in the response triggered by NBTXR3 + RT.

These results, along with the observed lymphocyte response, abscopal effect, and immunopeptidome changes, suggested that NBTXR3 + RT could impact TCR repertoire diversity in treated and untreated tumors. This possibility was confirmed by Darmon et al. [32]. In this study, the authors reported that the Simpson clonality, reflecting TCR diversity, was similar in treated and untreated tumors in control and NBTXR3 groups, with a slight, non-significant increase in the NBTXR3 group. In the RT group, treated and untreated tumors showed more variation. The NBTXR3 + RT group had significant differences between treated and untreated tumors and compared to the control. Treated tumors in the RT and NBTXR3 + RT groups also differed significantly. The Morisita similarity index was used to compare TCR repertoires. Control, NBTXR3, and RT groups had a high similarity, but the index dropped in the NBTXR3 + RT group, indicating increased TCR heterogeneity between treated and untreated tumors. Finally, expanded T-cell clone numbers in treated tumors were measured. The NBTXR3 group showed similar clone numbers to the control. RT and NBTXR3 + RT groups had significant increases in expanded clones compared to control. The NBTXR3 + RT group had more expanded clones than RT alone, but the difference was not statistically significant.

These combined findings suggest that NBTXR3 + RT, beyond cell destruction, can effectively modulate the adaptive antitumor immune response, robustly enough to trigger a systemic, distant response (abscopal effect).

### Combination of NBTXR3 with other therapeutic agents

RT is often used in combination with chemotherapy, and more recently with immunotherapies, to improve patient outcomes. However, these approaches have significant limitations. Chemotherapy, while effective, is often associated with substantial systemic toxicity, limiting its use in frail or elderly patients. Immunotherapies such as anti-PD-1 and anti-CTLA-4 have marked a major advancement in the treatment of certain cancers; however, their clinical benefit remains limited to a subset of patients due to innate or acquired resistance. Combining NBTXR3 with chemoradiotherapy could lead to better tumor responses while maintaining tolerable chemotherapy doses. Similarly, pairing NBTXR3 with RT and immunotherapy could boost the anti-tumor immune response by stimulating the release of tumor antigens and modifying the tumor microenvironment, potentially expanding the population of patients who respond to immunotherapy or overcoming resistance to immunotherapy. These combined approaches aim to leverage the synergies between different treatment modalities, providing new avenues to improve clinical outcomes in various types of solid tumors.

#### Chemotherapy

In vitro experiments on the human lung carcinoma NCI-H460-Luc2 cells showed that NBTXR3 + RT was as effective as cisplatin (CDDP) + RT in destroying these cells [17]. Addition of NBTXR3 to CDDP + RT improved cancer cell destruction. In vivo, experiments on NCI-H460 tumors revealed that both NBTXR3 + RT and CDDP + RT delayed tumor growth by about 22 days, compared to 17 days with RT alone. Notably, the combination of NBTXR3 + CDDP + RT was the most effective, extending tumor growth delay to approximately 25 days. Histological analysis of tumor tissues indicated that NBTXR3 + RT reduced cell proliferation and induced apoptosis, similar to the effects observed with CDDP + RT. Overall, this study indicates that NBTXR3 has the potential to improve the effectiveness of standard chemoradiation therapy in cancer treatment. While chemoradiotherapy remains the standard of care for several indications, these findings suggest that patients who are unable to receive chemotherapy may nonetheless benefit from this approach; a phase III clinical trial with JNJ-90,301,900 (NBTXR3) combined with RT ± cetuximab in elderly, platinum-ineligible patients with locally advanced head and neck squamous cell carcinoma (NCT04892173) is currently ongoing [45]. Furthermore, these findings open new possibilities for combining NBTXR3 with other chemoradiotherapies, such as poly(ADP-ribose) polymerase inhibitors (PARPi) or tyrosine kinase inhibitors (TKI).

#### Immunotherapy

Building on previous research, it was hypothesized that NBTXR3 combined with RT could overcome resistance to ICIs. Recent studies tested this approach using a dual-tumor model with the 344-SQR mouse lung cancer line, which is resistant to anti-PD-1 therapy [15, 16, 46, 47]. NBTXR3 was injected into one tumor, followed by targeted RT and various ICIs. An initial study tested whether the triple combination (NBTXR3 + RT + anti-PD-1) could overcome anti-PD-1 resistance and enhance the abscopal effect [16]. The triple combination significantly delayed growth in both irradiated primary and unirradiated secondary tumors compared to other treatments. It also remodeled the immune microenvironment of unirradiated tumors, activating innate and adaptive immune pathways, increasing CD8 + T cell infiltration, and expanding specific T cell clones, as shown by TCR repertoire analysis. This therapy reduced spontaneous lung metastases and enhanced antigen processing, T cell function, and trafficking, indicating a robust systemic and adaptive antitumor immune response.

A second study evaluated NBTXR3 + RT combined with anti-PD-1 and dual LAG3/TIGIT blockade [46]. The combination of NBTXR3 + RT with anti-PD-1, anti-LAG3, and anti-TIGIT markedly improved tumor control and survival, effectively suppressing both irradiated primary and unirradiated secondary tumors, with complete elimination in ~ 30% of mice. This efficacy was linked to robust immune activation, including increased infiltration and proliferation of CD8+, CD4+, and NK cells, as well as upregulation of genes involved in innate and adaptive immunity, antigen presentation, and interferon signaling. The therapy also induced durable immunological memory, as surviving mice resisted tumor rechallenge. Depletion studies confirmed that CD8 + and CD4 + T cells were essential for the anti-tumor effects, while NK cells played a lesser role.

The authors also evaluated NBTXR3 combined with immunoradiotherapy using NF-aCTLA4 and anti-PD-1 [47]. Adding NBTXR3 enhanced RT’s local effects by promoting cytotoxic T cell infiltration and immune gene activation in both primary and secondary tumors. NF-aCTLA4 outperformed conventional anti-CTLA4 by reducing Treg populations and boosting CD8 + T cell activity. The strongest results were achieved with the quadruple therapy (NBTXR3 + RT + NF-aCTLA4 + anti-PD-1), yielding 100% and 75% complete remission of primary and secondary tumors, respectively. This regimen amplified innate immune gene expression, suppressed immunosuppressive pathways, and enhanced cytotoxic lymphocyte recruitment and function. Responding mice exhibited long-term antitumor immunity, indicating durable immune memory.

The authors also evaluated the efficacy of combining NBTXR3 with high-dose (HDXRT, 3 × 12 Gy) and low-dose radiation therapy (LDXRT, 2 × 1 Gy) plus ICIs [15]. In this study, the primary tumor received high-dose radiation (HDXRT) with NBTXR3, while the secondary tumor received low-dose radiation (LDXRT). Combining NBTXR3 with HDXRT, LDXRT, and anti-PD-1/anti-CTLA4 slowed tumor growth, reduced lung metastases, and improved survival, with some mice achieving complete tumor eradication. Mechanistically, the therapy increased CD8 + T cell infiltration, decreased Tregs, enhanced immune-related gene pathways, and reshaped the TCR repertoire toward shared tumor-specific clonotypes. Treated mice also developed durable antitumor memory, rejecting tumor rechallenges.

Two recent articles evaluated the potential of combining NBTXR3 with proton beam therapy (PBT) and anti-PD-1 immunotherapy [48, 49]. Results indicated that PBT alone delayed primary tumor growth and produced an abscopal effect [48]. The addition of anti-PD-1 enhanced these effects, and the triple therapy (NBTXR3 + PBT + anti-PD-1) further improved tumor control at both sites. Mechanistically, it increased CD8 + T cell infiltration, activated cytotoxic pathways, and upregulated key anti-tumor genes, as shown by RNA and scRNA-seq analyses. Secondary tumors exhibited strong engagement of innate and adaptive immune cells, with reshaped microenvironments, including modulated neutrophils and increased M1 macrophages. Complete responders developed robust antitumor memory, rejecting tumor rechallenges, accompanied by enhanced dendritic cell activation and interferon-gamma production [49]. These data suggest that the unique physical properties of protons, combined with the radioenhancement effects of NBTXR3 and anti-PD1, may lead to better activation of the innate and adaptive immune systems.

## Conclusion

NBTXR3 is a radioenhancer specifically engineered to enhance the efficacy of radiotherapy by generating reactive oxygen species (ROS) within tumor cells (Fig. 2). In vivo studies have shown that NBTXR3 + RT significantly enhances tumor control and improves survival compared to RT alone. NBTXR3’s nanoparticles have also been found to increase lipid peroxidation, modulate the cancer cell immunopeptidome, induce immunogenic cell death, and enhance immune cell infiltration in tumors. Furthermore, NBTXR3 has exhibited effects when combined with immune checkpoint inhibitors, notably restoring anti-PD-1 efficacy in a resistant model and generating long-lasting antitumor memory responses. This ability to overcome resistance to anti-PD-1 represents a significant advancement in the field of cancer treatment. The properties of NBTXR3 allow for targeted amplification of radiation effects within tumor cells while simultaneously stimulating the immune system to mount a more effective anti-tumor response (Fig. 3).

By bridging the gap between radiotherapy and immunotherapy, NBTXR3 could potentially offer a new therapeutic option for those who have exhausted conventional ones.

## Data Availability

Not applicable.

## Abbreviations

CDDP: Cisplatin
DSB: DNA double-strand break
HDXRT: High-dose radiation therapy
HMGB1: High-mobility group box 1
ICD: Immunogenic cell death
ICI: Immune checkpoint inhibitors
IR: Ionizing radiation
IT: Intratumoral injection
LDXRT: Low-dose radiation therapy
LMP: Lysosomal membrane permeabilization
MN: Micronucleus
NK: Natural-Killer cells
NSCLC: Non-small cell lung cancer
PBT: proton beam therapy
PDX: Patient derived xenograft
ROS: Reactive oxygen species
RT: Radiotherapy
TCR: T cell receptor
TEM: Transmission electron microscopy
Treg: Regulatory T cell
